# Prevalence, Implications, and Risk Factors of Traumatic Dural Tears in Thoracic and Lumbar Fractures: A Retrospective Study

**DOI:** 10.7759/cureus.64351

**Published:** 2024-07-11

**Authors:** Nasrul Hanif Mohamad, Azizul Akram Salim, Mohd Imran Yusof, Phaik Shan Khoh, Han Sim Lim, Zairul Bahrin, Abdul Nawfar Sadagatullah

**Affiliations:** 1 Department of Orthopaedics, Universiti Sains Malaysia School of Medical Sciences, Kubang Kerian, MYS; 2 Department of Orthopaedics, Hospital Universiti Sains Malaysia, Kota Bharu, MYS; 3 Department of Orthopaedics, Hospital Sultanah Bahiyah, Alor Setar, MYS; 4 Department of Orthopaedics, Hospital Pulau Pinang, George Town, MYS

**Keywords:** spinal canal encroachment, laminar fracture, neurological deficit, traumatic dural tear, thoracolumbar fractures

## Abstract

Introduction

Spine fracture in association with traumatic dural tear is a serious injury. A traumatic dural tear is difficult to determine based on initial clinical presentation and radiological imaging even with magnetic resonance imaging (MRI). However, during decompression surgery, cerebrospinal fluid leaks surrounding the injured segments are usually confirmed by directly visualizing them. For preoperative planning and intraoperatively limiting further damage to the dural and neurological structures, early detection of traumatic dural tears before surgery is important. This study aims to determine the prevalence, implication, risk factors, and complications of traumatic dural tears in patients who have undergone surgical treatment for thoracic and lumbar fractures. We believe our retrospective study would identify more accurate risk factors for traumatic dural tears and aid us with preoperative planning and operative precaution.

Methods

This study retrospectively included all patients who had thoracic and lumbar fractures and had posterior instrumentation and decompression surgery at three hospitals in the Northern region of Malaysia from January 2018 to December 2020. Fractures associated with pathological spine including metastatic, severe osteoporosis, ankylosing spondylitis, metabolic bone disease, those with missing data, and iatrogenic dural tears were excluded from this study. Preoperative and postoperative neurological assessments based on the American Spinal Injury Association (ASIA) impairment scale, blood loss volume, duration of the surgery, and post-surgery complications were gathered from medical records. Interpedicular distance, ratio of central canal diameter, laminar fracture gap, and pedicle fractures were identified and measured. The obtained data was analyzed using Pearson's chi-square and Fisher's exact test for categorical variables, and independent t-test/Mann-Whitney test for numerical variables.

Result

This study comprised a total of 93 patients who had fractures in their thoracic and lumbar regions. The mean age of the patients was 38 years. The number of patients with traumatic dural tears was 20 (21.5%). There was an association between the presence of dural tears and preoperative neurological deficits (P<0.001). Wider mean interpedicular distance (P=0.004), increased central canal diameter ratio (P<0.001), and displaced laminar fracture (P<0.001) were significantly higher in patients with traumatic dural tears. Multiple logistic regression analysis showed both incomplete (P=0.002) and complete (P=0.037) preoperative neurological deficit, increase of central diameter ratio of encroachment (P=0.011), and presence of >2mm laminar fracture gap (P=0.015) had a significant association with a traumatic dural tear. This study found that patients with traumatic dural tears had longer surgical times and statistically larger mean blood loss volumes when compared to patients without dural tears (P<0.001). There is no significant association between the complications following the surgery and the presence of a dural tear (P>0.05).

Conclusion

This study shows that the presence of preoperative neurological deficits, wider interpedicular distance, severe canal encroachment, and wide separation of laminar fracture may indicate the likelihood of traumatic dural tear in spine fracture. These factors will enable surgeons to enhance their operational planning and make early preparations for the management of dural tears.

## Introduction

Spinal fractures may lead to traumatic dural tears, leading to leakage of cerebrospinal fluid (CSF). The diagnosis of traumatic dural tears is challenging to establish using clinical and radiological means, including magnetic resonance imaging (MRI), before surgery. Most of the time it is identified during decompression surgery and posterior instrumentation by visualization of CSF leakage around the injured segments [1,2].

Several researchers have documented that a burst fracture accompanied by a vertical laminar fracture is primarily linked to a dural tear. Nevertheless, traumatic tears are also observed in other spinal injuries, specifically in cases with unstable injuries [3,4]. Several publications have looked at predicted factors for dural tears, the preoperative neurological status related to them, and the morphological pattern of the injured spinal canal in thoracolumbar fractures. However, most of these studies have mostly focused on thoracic and lumbar burst fractures. Currently, there is a scarcity of information that specifically addresses traumatic dural tear in other type of spine fractures [4-6].

Managing traumatic spine injuries with dural tears is still a challenging undertaking. Determining the presence of a dural tear before surgery can be difficult due to the lack of sensitive imaging tools that can detect microscopic tears or slight leakage of CSF. A traumatic spinal dural tear can result in the avulsion of nerve roots or the entrapment of neural elements in the fracture. This might lead to a neurological deficit that requires immediate decompression surgery. Several studies have documented various consequences associated with dural laceration, such as CSF leakage resulting in the formation of a pseudo meningocele, dura-cutaneous fistula, meningitis, epidural abscess, intracranial subdural hematoma, nerve root entrapment, wound healing issues, and chronic headache [7-12]. Therefore, early recognition of traumatic dural tears is essential for operative planning and preventing additional injuries to the dural and neural structures. This study aims to determine the prevalence, implications, and complications of traumatic dural tears in patients who have received surgical intervention for thoracic and lumbar fractures. Additionally, it aims to identify clinical and radiographic factors that may be related to dural tears.

## Materials and methods

This retrospective study included all patients with fractures in the thoracic and lumbar regions who underwent surgery with posterior instrumentation and decompression at three hospitals in the Northern region of Malaysia from January 2018 to December 2020. The clinical records and radiologic data of these patients were reviewed and gathered using a pro forma checklist. Patients with thoracic and lumbar fractures associated with underlying pathological spine disease, including metastatic spine disease, severe osteoporosis, ankylosing spondylitis, bone tumor, metabolic bone disease, those with missing data, and iatrogenic dural tear were excluded from this study. The study was approved by the Human Research Ethics Committee Universiti Sains Malaysia (approval number: USM/JEPeM/21070500). This study evaluated complications resulting from traumatic dural tears that occurred over three years, including persistent CSF leak, pseudomeningocele, meningitis, and the need for additional surgery.

Data collection

The data collected included biodemography, radiological assessment, measurements from radiographs, CT imaging, and intraoperative findings based on a computerized operating documentation System (COTDS). We collected all relevant data on the intraoperative records such as the existence of pedicle or laminar fracture, the duration of the surgery, and any complications related to traumatic dural tear from the COTDS. Diagnosis of traumatic dural tear was confirmed by direct visualization of CSF leakage or nerve root entrapment intraoperatively by the same surgeon. If a dural tear was detected, the operating surgeon would measure its length and proceed to repair it.

Evaluation process

We applied the standard American Spinal Cord Injury Association (ASIA) impairment scale to evaluate the degree of motor and sensory impairment in patients with spinal cord injuries during the neurological status examination. The grading scale for injuries ranges from A to E, with A being the most serious injury and E representing the least severe. In terms of the radiological assessment, we identified the type of thoracic and lumbar fractures based on radiographs and CT imaging and classified them based on AO Spine Thoracolumbar Spine Injury Classification. The injury morphology is classified as an A injury (compression), B injury (distraction), or C injury (translation). Type A fractures were graded in increasing severity as follows: A0 (simple), A1 (compression), A2 (pincer), A3 (burst involving one end plate), and A4 (burst involving both endplates). Type B fractures included classic bony chance (B1), failure of the posterior tension band such as horizontal fracture lines through the posterior elements or evidence of posterior ligamentous disruption (B2), and hyperextension injuries (B3). Type C fractures/injuries demonstrate a dissociation between cranial and caudal segments. If more than one injury was evident, the most severe injury was recorded. Fracture level is divided into 4 groups which are upper thoracic (T1-T5), lower thoracic (T6-T10), the thoracolumbar junction (T11-L2), and lumbar (L3-L5).

**Figure 1:**
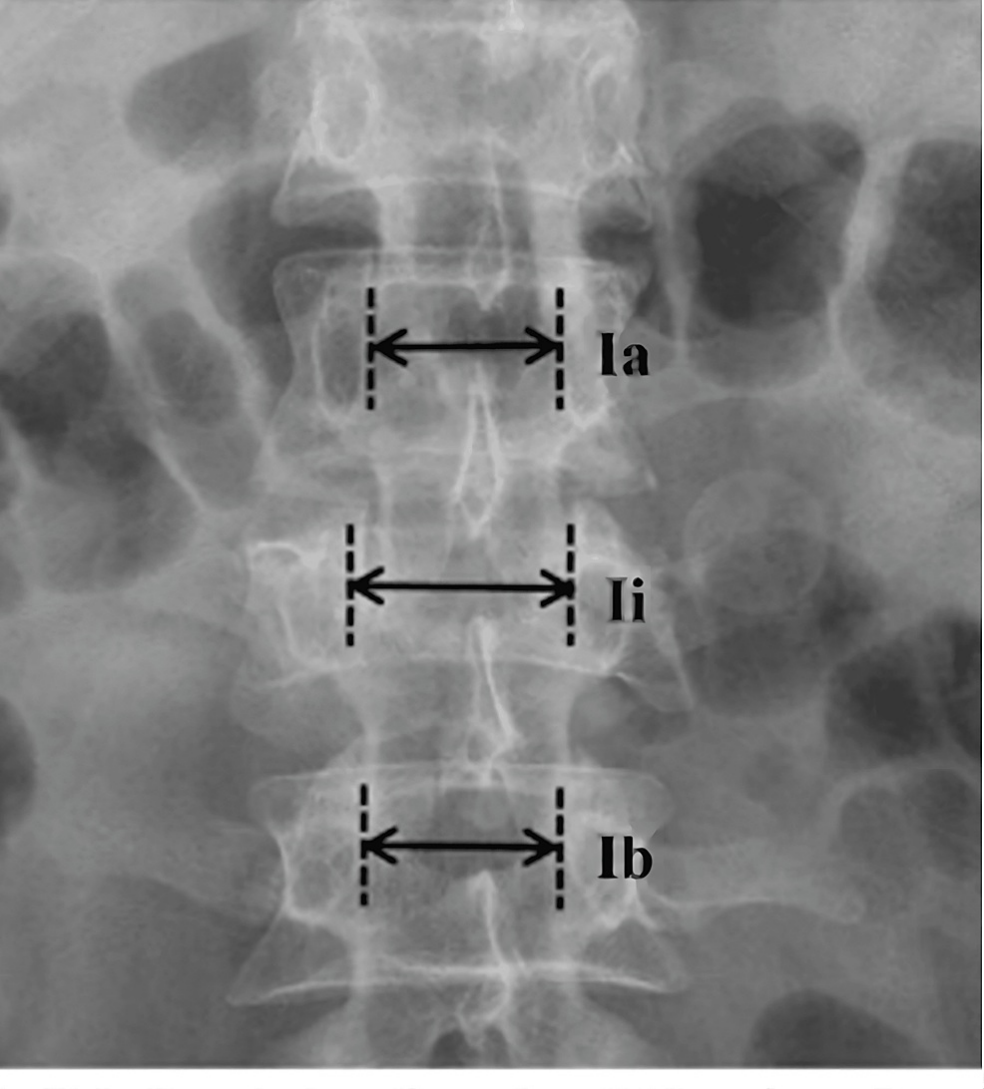
Interpedicular distance (IPD) is measured from an anteroposterior radiograph. It is measured from the medial sclerotic area of the pedicles at the level of fracture (1i). Normal IPD is defined as the average (In) of the corresponding distance above (1a) and below (1b) the injured level and calculated as (1a+1b)/2.

Each patient's thoracolumbar fracture level and pattern were assessed using available anteroposterior and lateral radiographs and CT. Interpedicular distance was measured from an anteroposterior radiograph. It was measured from the medial sclerotic area of the pedicles at the level of fracture (1i). Normal interpedicular distance (IPD) is defined as the average (In) of the corresponding distance above (1a) and below (1b) the injured level and calculated as (1a+1b)/2. IPD increment is calculated as 100% x (1i-In)/In (Figure 1).

**Figure 2:**
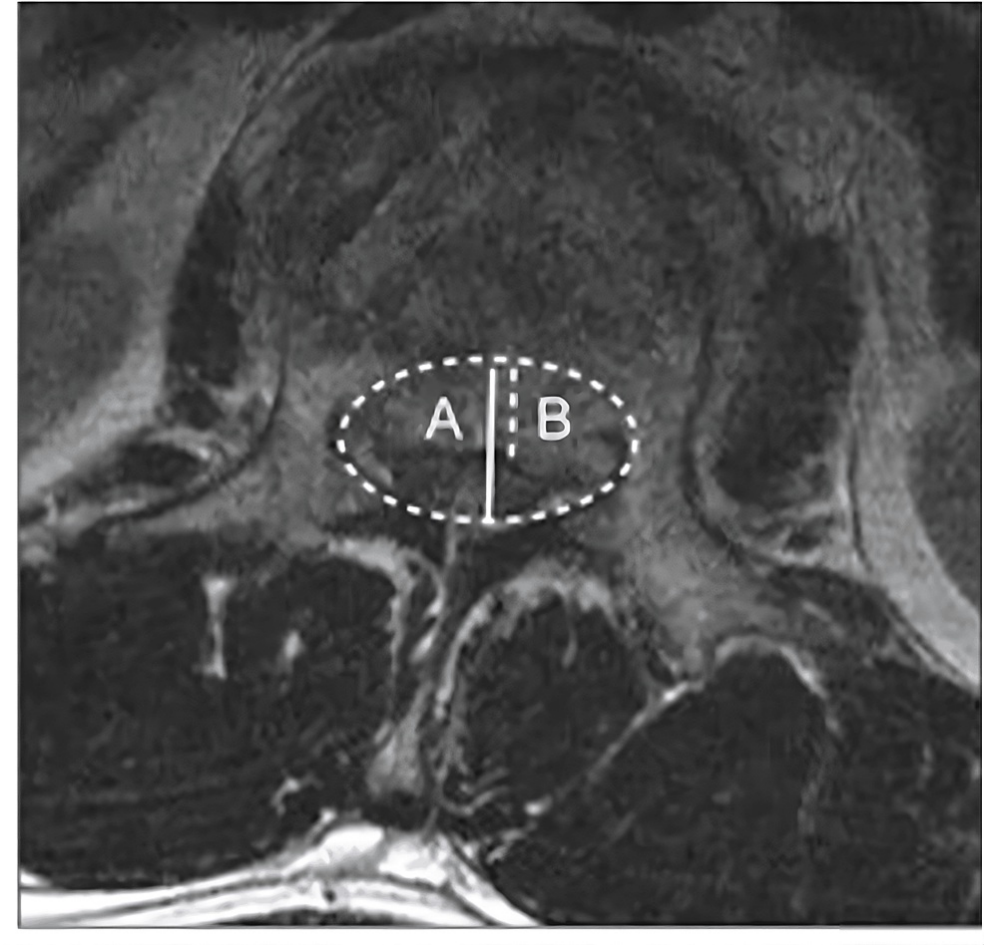
The neural canal encroachment ratio is measured by the ratio of retropulsion bony fragment anteroposterior diameter and central canal anteroposterior diameter in the CT axial plane.

The neural canal encroachment ratio was measured by the ratio of retropulsion bony fragment anteroposterior diameter and central canal anteroposterior diameter in the CT axial plane (Figure 2).

**Figure 3:**
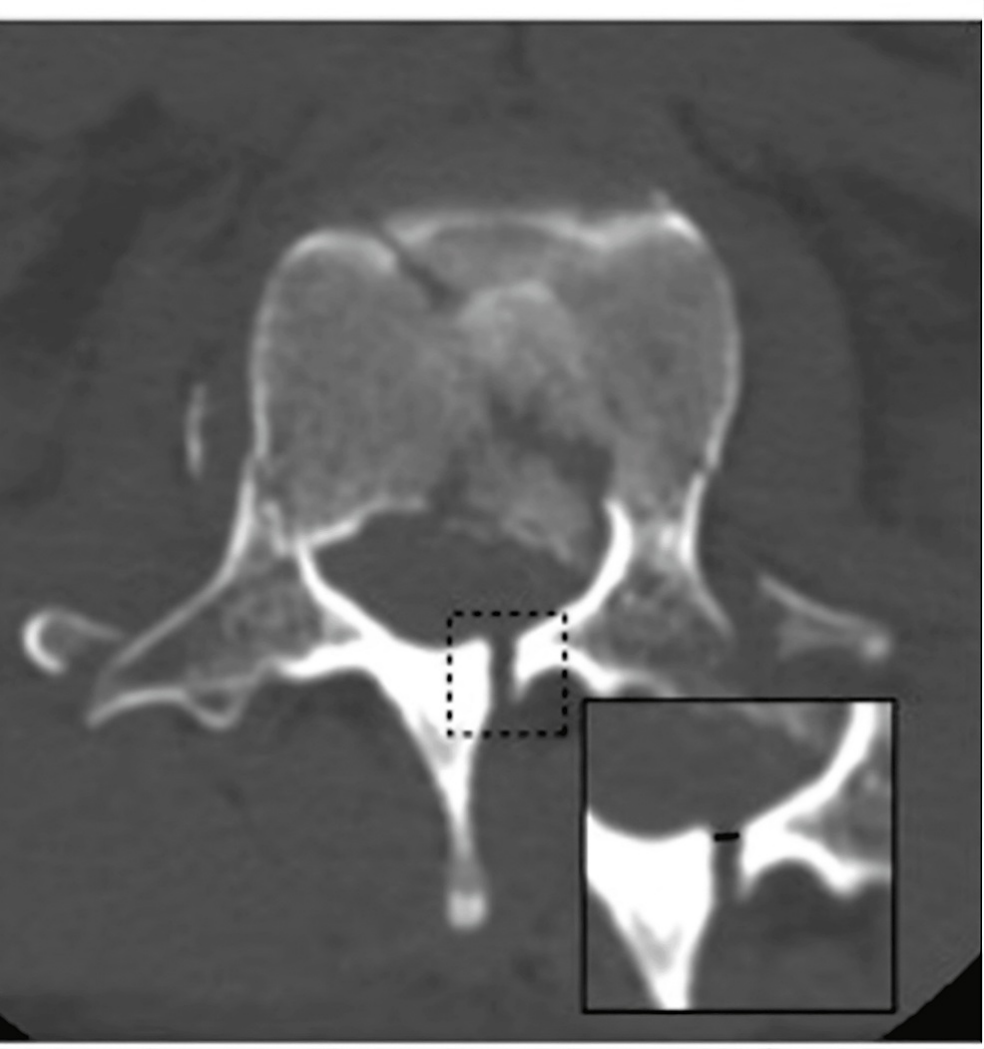
The presence of laminar and pedicle fracture is identified and the distance of the separated lamina is measured in CT axial plane.

The presence of laminar and pedicle fracture is identified and the distance of the separated lamina was measured in the CT axial plane (Figure 3).

Statistical analysis

The obtained data was analyzed using Pearson's chi-square and Fisher's exact test for categorical variables, and independent t-test/Mann-Whitney test for numerical variables. The analysis was based on the patients' medical records. All information was recorded in an MS Excel worksheet (Microsoft Corporation, Redmond, Washington, United States) and exported into IBM SPSS Statistics for Windows, Version 24.0 (Released 2016; IBM Corp., Armonk, New York, United States) for analysis.

## Results

This study included 93 patients who had undergone surgical treatment for thoracic and lumbar fractures at hospitals in the Northern region of Malaysia. The mean age of the patients was 38 years (Table 1). Seventy-four (79.6%) patients were male and 19 (20.4%) were female. Regarding ethnicity, 68 (73.1%) were Malay, 13 (14.0%) were Chinese, and seven (7.5%) were Indian. A total of 57 (61.3%) had motor vehicle accidents (MVA) and 36 (38.7%) had fallen from height.

**Table 1:** Baseline characteristics of the study population (N=93)

Variable	Values
Age (years), mean±SD	38.14±13.66
Sex, n (%)	
Female	19 (20.4)
Male	74 (79.6)
Ethnicity, n (%)	
Malay	68 (73.1)
Chinese	13 (14.0)
Indian	7 (7.5)
Others	5 (5.4)
Mechanism of injury, n (%)	
Fall from height	36 (38.7)
Motor vehicle accident	57 (61.3)

Table 2 shows the proportion of traumatic dural tears in patients with thoracic and lumbar fracture surgery at the hospitals involved. Overall, the prevalence of patients with traumatic dural tears was 21.5% (20 patients).

**Table 2:** Proportion of traumatic dural tears in the study population (N=93)

Dural tear	Frequency (percentage)
Yes	20 (21.5)
No	73 (78.50

Table 3 shows the descriptive statistics for each subgroup based on traumatic dural tears. The proportion of those with MVA mechanism injury with traumatic dural tears was 12 patients (60.0%) The findings indicate that there was an association between the dural tear and preoperative neurological deficit (P<0.001). A total of seven patients (35.0%) with an incomplete status were among those with traumatic dural tears. Based on the imaging parameters, the mean interpedicular distance, the ratio of central canal diameter, and laminar fracture were significantly higher in patients with traumatic dural tears, with 5.28, 0.69, and 2.99, respectively (P< 0.05).

**Table 3:** The descriptive statistics of factors associated with the presence of traumatic dural tears in the study population (N=93) ^a^Pearson chi-square applied; expected count less than 5 < 20% ^b^Fisher exact test applied; expected count less than 5 > 20% ^c^Independent t-test applied; normality and equal variance assumptions were met Data is presented as n (%) and mean±SD

Variable	Dural Tear (n=20), n (%)	No Dural Tear (n=73), n (%)	Total (n=93), n (%)	P-value
Mechanism injury				
Fall from height	8 (40.0)	28 (38.4)	36 (38.7)	0.894^a^
Motor vehicle accident	12 (60.0)	45 (61.6)	57 (61.3)	
Preoperative neurological deficit				
Complete	11 (55.0)	10 (13.7)	21 (22.6)	<0.001^b^
Incomplete	7 (35.0)	14 (19.2)	21 (22.6)	
Normal	2 (10.0)	49 (67.10	51 (54.8)	
Fracture level				
Upper thoracic	2 (100.0)	14 (19.2)	16 (17.2)	0.186^b^
Lower thoracic	0 (0.0)	10 (13.7)	10 (10.8)	
Thoracolumbar junction	16 (80.0)	41 (56.2)	57 (61.3)	
Lumbar	2 (10.0)	8 (11.0)	10 (10.8)	
Morphological pattern of fracture (AO TL Spine Injury Classification)				
Type A	16 (80.0)	55 (75.3)	71 (76.3)	0.911^b^
Type B	2 (10.0)	11 (15.1)	13 (14.0)	
Type C	2 (10.0)	7 (9.6)	9 (9.7)	
Imaging parameters				
Interpedicular distance, mean±SD	5.28±3.05	3.25±2.62	3.69±2.83	0.004^c^
Ratio of central canal diameter, mean±SD	0.69±0.14	0.41±0.19	0.46±0.21	<0.001^c^
Laminar fracture, mean±SD	2.99±1.12	1.12±1.66	1.53±1.73	<0.001^c^
Pedicle fracture				
No	10 (50.0)	24 (32.9)	34 (36.6)	0.159^a^
Yes	10 (50.0)	49 (67.1)	59 (63.40)	

Table 4 shows the application of simple and multiple logistic regression in predicting the factors associated with traumatic dural tears among patients who have undergone surgical treatment. All variables with P-value <0.25 from simple logistic regression were included in multiple logistic regression. Variable selection was performed using forward and backward selection. The results indicate that preoperative neurological deficit, central canal diameter ratio, and presence of laminar fracture showed a statistically significant association with a traumatic dural tear in multiple logistic regression.

**Table 4:** Associated factors of the presence of traumatic dural tears in the study population (N=93) ^a^Simple logistic regression applied ^b^Backward stepwise multiple logistic regression model applied The model’s overall fit was checked and reported to be the Hosmer-Lemeshow test (P=0.859), overall, correctly classified percentage=88.2%, and area under the curve (94.2%) was applied to check the model fitness.

Variables	Simple logistic regression		Multiple logistic regression	
	Crude OR (95% CI)	P-value^a^	Adjusted OR (95% CI)	P-value^b^
Mechanism injury				
MVA	0			
Fall from height	1.071 (0.390, 2.946)	0.894		
Preoperative neurological deficit				
Normal	0		0	
Incomplete	26.950 (5.160, 140.753)	<0.001	22.332 (2.986, 167.001)	0.002
Complete	12.250 (2.283, 65.726)	0.003	8.674 (1.134, 66.364)	0.037
Fracture level				
Upper thoracic	0			
Lower thoracic	0.000 (0.000, 0.000)	>0.95		
Thoracolumbar junction	2.732 (0.557, 13.399)	0.216		
Lumbar	1.750 (0.205, 14.931)	0.609		
Morphological pattern of fracture				
Type C	0			
Type B	1.018 (0.192, 5.393)	>0.95		
Type A	0.636 (0.072, 5.613)	0.684		
Interpedicular distance	1.267 (72.875, 206014.80)	0.007		
Ratio of central canal diameter	3874.71 (72.875, 206014.795)	<0.001	846.113 (4.808, 148891.210)	0.011
Laminar fracture				
≤2mm	0		0	
>2mm	15.017 (3.968, 56.824)	<0.001	7.537 (1.481, 38.372)	0.015
Pedicle fracture				
No	0			
Yes	2.042 (0.749, 5.569)	0.163		

The final model for associated factors with the presence of traumatic dural tears among patients who have undergone surgical treatment for thoracic and lumbar fractures using multiple logistic regression could be interpretable as (i) Patients with incomplete preoperative neurological deficits have an increased risk of traumatic dural tear by 22.332 compared to patients with normal preoperative neurological functions (Adjusted OR (aOR) =22.332; 95%CI: 2.986, 167.001; P=0.002), (ii) Patients with complete preoperative neurological deficits have an increased risk of traumatic dural tears by 8.674 compared to patients with normal preoperative neurological functions (aOR=8.674; 95%CI: 1.134, 66.364; P=0.037), (iii) For every one-unit (mm) increase in encroachment of central canal diameter ratio, the risk of getting traumatic dural tear increased by 846.11 (aOR=846.113; 95%CI: 4.808, 148891.210; P=0.011), and (iv) Patients with a laminar fracture >2mm have an increased risk of traumatic dural tear by 7.537 compared to patients with a laminar fracture ≤2mm (aOR=7.537; 95%CI: 1.481, 38.372; P=0.015).

There was a significantly higher mean blood loss volume and duration of surgery in patients with traumatic dural tears than in those without traumatic dural tears (P<0.001) (Table 5). The mean±SD blood loss volume in those with and without dural tears was 1365.00±571.26) ml and 734.93±444.15 ml, respectively. The mean±SD duration of surgery among those with traumatic dural tears was 268.75±104.25 minutes.

**Table 5:** Comparison of blood loss and duration of surgery between patients with and without dural tear ^a^Independent t-test was applied; normality and equal variance assumptions were met

Variables	Dural Tear (n=20), mean±SD	No Dural Tear (n=73), mean±SD	Mean Difference (95% CI)	P-value^a^
Blood loss volume (ml)	1365.00±571.26	734.93±444.15	630.07 (392.68, 867.46)	<0.001
Duration of surgery (min)	268.75±104.25	168.56±71.14	100.19 (60.47, 139.91)	<0.001

There was a significant association between the presence of traumatic dural tears and the postoperative ASIA impairment scale (P<0.001) (Table 6). The results indicated that 11 patients (55.0%) with complete neurological deficits and seven patients (35.0%) with incomplete neurological deficits had traumatic dural tears. A total of 54 patients (74.0%) had normal neurological assessment in those without traumatic dural tear.

**Table 6:** Comparison of postoperative ASIA impairment scale between patients with and without dural tear in the study population (N=93) ^b^Fisher exact test applied; expected count more than 5 > 20% ASIA: American Spinal Cord Injury Association

Variables	Dural Tear (n=20), n (%)	No Dural Tear (n=73), n (%)	Total (n=93), n (%)	P-value^b^
Postoperative ASIA impairment scale				
Complete neurological deficit	11 (55.0)	11 (15.1)	22 (23.7)	<0.001
Incomplete neurological deficit	7 (35.0)	8 (11.0)	15 (16.1)	
Normal	2 (10.0)	54 (74.0)	56 (60.2)	

There was no significant mean difference in the duration of surgery time between the type of fracture level among those with traumatic dural tears (P>0.05) (Table 7). 

**Table 7:** Comparison of duration of surgery time based on fracture level among patients with traumatic dural tears in the study population (N=20) ^a^One-way ANOVA applied; normality and equal variance assumptions were met

Variable	Dural Tear (Upper Thoracic), mean±SD	Dural Tear (Lower Thoracic), mean±SD	Dural Tear (Thoracolumbar Junction), mean±SD	Dural Tear (Lumbar), mean±SD	P-value^a^
Duration of surgery time (minutes)	225.00±106.07	270.31±110.34	300.00±84.85	268.75±104.35	0.785

When comparing the length of dural tear repair based on the postoperative ASIA impairment scale, it had no significant mean difference in the length of dural tear between the postoperative ASIA impairment scale among those with traumatic dural tears (P>0.05) (Table 8).

**Table 8:** Comparison of length of dural tear based on postoperative ASIA impairment scale among patients with traumatic dural tears in the study population (n=20) ^a^One-way ANOVA applied; normality and equal variance assumptions were met ASIA: American Spinal Cord Injury Association

Variables	Postoperative ASIA impairment scale			
	Complete neurological deficit (n=11), mean±SD	Incomplete neurological deficit (n=7), mean±SD	Normal (n=2), mean±SD	P-value^a^
Length of dural tear (mm)	24.00±8.10	31.67±14.72	27.50±3.54	0.397

Regarding complications, 76 (81.7%) out of the 93 total patients experienced no complications. Meanwhile, seven patients (7.5%) had sacral sores, one patient had implant failure (1.1%), one patient had passed away (1.1%), two patients had prominent screws, and six patients (6.5%) had a re-surgery or surgical site infection (Table 9).

**Table 9:** Proportion of complications attributable to traumatic dural tears in the study population (N=93)

Complications	Frequency (Percentage)
Sacral sore	7 (7.5)
Implant failure	1 (1.1)
Passed away	1 (1.1)
Prominent screw	2 (2.2)
Re-surgery/surgical site infection	6 (6.5)
Nil	76 (81.7)

## Discussion

Traumatic dural tear in spine fracture is uncommon but a potentially severe injury. Untreated, this condition can result in persistent CSF leaking, which can then lead to the development of pseudomeningocele, infection, meningitis, and potential harm to the neural structures. The surgeon always encounters challenges in identifying traumatic dural tears before surgery. The confirmation of CSF leakage can only be achieved with direct visualization during the decompression procedure and posterior instrumentation. Performing spinal reductions without identifying dural tears can lead to an extension of the rupture and potential injury to neurological structures [6].

Traumatic dural tear following thoracolumbar fracture was first described by Miller et al. [13]. In his case series, 18 out of 19 patients with unstable lumbar burst fractures had dural tears and entrapment of the nerve root at the injured segment. The incidence of traumatic dural tears in spine fractures reported in the literature ranges from 7.7% to 27.4% [3,5,14,15]. Our retrospective study found that the occurrence of traumatic dural tears among patients with thoracic and lumbar spine fractures in the northern region is approximately 21.5%, which is greater than what previous research has reported.

Identifying a traumatic dural tear before surgery is difficult as it cannot be accurately diagnosed by clinical or radiographic methods. Currently, there are no sensitive imaging techniques that can detect small dural tears or minor CSF leaks. The diagnosis of traumatic dural tear is primarily made during surgery by directly observing the leaking of CSF surrounding the area of injury. However, if the rupture is small and cannot be seen by MRI before the surgery, it is more likely to be missed by the surgeon [16]. 

Several publications have identified significant predictive indicators for traumatic dural tears in thoracic and lumbar fractures based on clinical and radiographic data. Aydinli et al. found no significant difference in respect of etiologies with the occurrence of dural tears in thoracic and lumbar fractures [3]. According to other authors, dural tears are found equally at low thoracic and lumbar levels [3,17]. Argenson et al. [18] reported that they found a greater frequency in the lumbar area while other authors like Camissa et al. [19] saw dural tears only in lumbar burst fractures. There was an association between the dural tear and preoperative neurological deficit (P<0.001). A total of seven patients (35.0%) with incomplete neurology and 11 patients (55.0%) with complete neurology were among those with traumatic dural tears. Most of the traumatic dural tears patients were classified as Type A (80%), and the rest were classified as Type B (10%), and Type C (10%). Neurological impairment has been observed in 30-60% of individuals with thoracolumbar burst fractures. However, Keene et al. have argued that the compromised neural canal resulting from the intrusion of bone does not necessarily correspond to the neurological condition of the patient [15].

The neurological consequences of fractures and dislocations in the thoracic and lumbar spine are believed to be more closely associated with the site and type of injury, rather than the percentage of neural canal impacts. Cammisa et al. found that a preoperative neurological deficit, when combined with a lumbar burst fracture and laminar fracture, was a highly sensitive (100% sensitivity, 74% specificity) indicator of dural tears [18]. It was determined that patients who had a neurological deficit and a laminar fracture along with a lumbar burst fracture were more likely to experience dural tears and entrapment of neural elements. Other authors also suggested a significant association between the presence of dural tears and a severe neurological deficit. They emphasized that for patients who have laminar fractures with neurological deficits, the possibility of dural tears should be a major concern [20]. In another study, the mean distance of the separated lamina was 2.4±1.2 mm and 1.0±0.6 mm between the group of burst fractures and laminar fractures with dural tear and the group of burst fractures with laminar fracture without a dural tear (P-value =0.002). It showed a statistically significant difference between the two groups. The potential of damage resulting from a displaced dura mater will be higher when there is an increased distance between the lamina and sharp edges [6]. However, in our study, fracture level injury (P-value 0.186) and morphological pattern of thoracic and lumbar fracture (P-value 0.911) did not show statistical significance.

Based on the imaging parameters, the mean interpedicular distance, the ratio of central canal diameter, and laminar fracture were significantly higher in patients with traumatic dural tears. ﻿The interpedicular distance (IPD) increment was also an important predictive factor of dural tears in our study. The mean interpedicular distance increment was 5.28±3.05 mm in patients with dural tears and 3.25±2.62 mm in those who did not have dural tears (P-value 0.004). ﻿For burst fractures, previous studies have reported significant changes in the IPD in clinical series. Fernanda et al. found a significant ﻿ correlation between IPD and the percentage ﻿of narrowing of the spinal (P-value 0.000) [21]. ﻿IPD was significantly increased in patients with ﻿a neurological deficit (24.7% ± 12.6%) and in patients with lamina ﻿fractures (24.6% ± 16.2%), similar factors that were reported in our study. Other authors both reported that wider interpedicular distance in the burst fractures was an independent risk factor for traumatic dural tears [5,6].

By doing multiple logistic regression analyses, we have discovered four significant findings about the factors associated with traumatic dural tears: (i) Patients with incomplete preoperative neurological deficit had an increased risk of traumatic dural tear by 22 times compared to patients with normal preoperative neurological functions (aOR=22.332; 95%CI: 2.986, 167.001; P=0.002), (ii) Patients with complete preoperative neurological deficit had increased risk of traumatic dural tear by 8.5 times compared to patients with normal preoperative neurological functions (aOR=8.674; 95%CI: 1.134, 66.364; P=0.037), (iii) For every one-unit (mm) increase in central canal diameter ratio, the risk of getting traumatic dural tear increased by 846 times (aOR=846.113; 95% CI: 4.808, 148891.210; P=0.011), and (iv) Patients with laminar fracture >2mm had increased risk of traumatic dural tear by 7.5 times compared to patients with a laminar fracture ≤2mm (aOR=7.537; 95%CI: 1.481, 38.372; P=0.015).

A previous study done by Park et al. revealed that preoperative neurological deficit, wider interpedicular distance, wider separation of the laminar fracture, and severe canal encroachment might be predictable factors for traumatic dural tear in thoracolumbar fractures [6]. ﻿Nevertheless, the sample size in their study was small (n=31) for analysis. Another study done by Xu et al with a bigger sample size (n=113) reported that the incidence of dural tears was 27.4% [5]. They also found that patients with dural tears had significantly worse preoperative neurological status, wider interpedicular distance, greater separation of laminar fractures, and larger encroachment of retropulsed fragments in the bony spinal canal. They identified two independent risk factors for dural tears: ﻿the ratio of the interpedicular distance of more than 125% and the encroachment of retropulsion fragments ratio in the bony spinal canal of more than 50%. The ratio of retropulsion fragments encroaching into the bony spinal canal was determined to be the most important risk factor for dural tears. The epidural fat and ligamentum flavum may be crucial components of the dural protective mechanism. An intrusion of retropulsed fragments may cause a dural sac to protrude into the ligamentum flavum or the epidural fat. At the time of trauma, the anamorphic dural sac is protected by the ligamentum flavum, which includes elastic fibers, and epidural fat. Additionally, the dural sac may be able to be accommodated by the foramen. Due to a lack of buffers, there is an increased risk of dural entrapment and laceration in cases when fragments invade the bone canal. Maximal separation of laminar fracture was not shown to have an association with an increased risk of dural laceration [5]. However, our study showed that patients with laminar fractures>2mm increased the risk of traumatic dural tears by 7.5 times compared to patients with laminar fractures ≤2mm. 

Most of our traumatic dural tears occurred at the level of the thoracolumbar junction (n=16) and lumbar (n=2) regions and only two patients occurred in the upper thoracic region. The thoracic and lumbar spine can be divided into three regions, namely, the thoracic (T1-T10), the thoracolumbar junction (T10-L2), and the lumbar (L3-L5). The thoracic spine is rigid due to the coronal orientation of facet joints, narrow intervertebral disc, and the presence of ribcage. ﻿Thus, it requires huge amounts of energy to produce fractures and dislocations. The narrow spinal canal in this region predisposes spinal cord damage resulting in a high incidence of neurological deficit. The lumbar spine is relatively flexible due to the thicker intervertebral discs, the more sagittal orientation of facet joints, and the absence of rib cages. The relatively lesser incidence of neurological injury in lumbar fractures can be attributed to the large size of the neural canal and the greater resilience of the cauda equina nerve roots. The thoracolumbar junction (T11-L2) is uniquely located between the rigid thoracic and mobile lumbar spines. This transition from the less mobile thoracic spine with its associated ribs and sternum to the more dynamic lumbar spine subjects the thoracolumbar region to significant biomechanical stress. Hence, fractures of the thoracolumbar region are the most common injuries in the vertebral column [22]. 

To the best of our knowledge, no prior study has analyzed the effects of traumatic dural tears on surgical duration, intraoperative blood loss, and postoperative neurological recovery in patients who undergo dural injury repair compared to those without such repair. The only studies available for comparison were those involving iatrogenic dural tears. We found that there was a significantly higher mean blood loss and duration of surgery in patients with traumatic dural tears than in those without traumatic dural tears (P<0.001). The mean±SD blood loss volume in those with and without dural tears was 1,365.0±571.26 ml and 734.9±444.15 ml. The mean±SD duration of surgery among those with traumatic dural tears was 268.8±104.25 minutes. The correlation between an extended period of surgery and an increased volume of blood loss in patients with traumatic dural tears can be attributed to the additional surgery necessary for repairing the dural tear in addition to decompression and posterior instrumentation. Traumatic dural tear is typically more intricate than iatrogenic dural tear. Identifying a dural tear can be tough, and repairing it requires careful attention to detail due to the nature of the tear. ﻿The frequently utilized agents for facilitating the repair process include sutures, fibrin sealants, and commercial collagen matrix grafts, either used individually or in conjunction with other repair procedures. Less often employed substances include fascia, adipose tissue graft, oxidized cellulose polymer products, muscle tissue, and absorbable gelatin sponges [14]. We usually use Prolene 6/0 sutures (Ethicon, Inc., Raritan, New Jersey, United States) and a dural patch or Lyoplant (Aesculap, Inc., Pennsylvania, United States) for the repair.

In our study, all patients with traumatic dural tears with complete and incomplete preoperative neurological deficits did not show significant improvement in neurological recovery three months post dural repair (P<0.001). Takenaka et al. demonstrated that dural tear was associated with a higher incidence of postoperative surgical site infection, postoperative neurological deficit, and postoperative delirium, in addition to direct dural tear-related dural leak [23]. ﻿We cannot conclude a causative relationship between dural tears and postoperative neurological deficits using the results of our study and previous reports. Nonetheless, the correlation between these two complications implies that neuronal components could sustain damage or become entrapped when the dura tears during the initial trauma. Our study showed no significant association of duration of surgery time between the types of fractures among those with traumatic dural tears (P>0.05) and no significant difference in length of dural tears between the postoperative ASIA impairment scale among those with traumatic dural tears (P>0.05). 

Luszczyk et al. demonstrated an overall direct complication rate of 2.1% in patients who sustained traumatically induced dural tears as a result of spine injuries [14]. Two patients developed pseudomeningocele (one managed surgically and one medically), and two patients developed persistent CSF drainage, both of which required additional operative management. An additional 5.3% of patients required irrigation and debridement secondary to wound infection, but no persistent CSF leak was identified and therefore causality was difficult to determine. However, the infection rate in their series appears favorable compared with reported postoperative infection rates in the setting of operatively managed spine trauma, which has been reported at a rate of 10-13% [24,25]. The negative impact of traumatic dural tears on the patient’s postoperative complication appears to be low and similar to the lower end of complication rates reported for iatrogenically created dural tears in elective spine surgery [14]. Adverse events related to dural tears include persistent CSF leak leading to pseudomeningocele, dura-cutaneous fistula, wound healing complications, meningitis, arachnoiditis, epidural abscess, and persistent headache [7,8,9,10]. In our study, 81.7% had no complications, seven patients (7.5%) had sacral sores, and six patients (6.5%) had to undergo another surgery or had surgical site infection.

There were several limitations that we encountered during our study. A limited number of patients with traumatic dural tears in thoracic and lumbar fractures, as observed in our retrospective data collection, have a substantial influence on our data analysis. Despite the multicenter setting of this investigation and the three-year data-collecting period, we obtained only a limited number of samples due to the low occurrence of spine fractures with traumatic dural tears in the region. We encountered difficulties during the process of data collecting, particularly about the patient's records. Although we employed standard procedures for repairing the injured dura and implemented suitable rehabilitation measures after the surgery, our investigation revealed no enhancement in neurological recovery among our patients three months post dural repair. It may be asserted that patients with neurological impairment, regardless of whether they had a dural injury or not, would not see recovery within three months after surgery. Prolonged surveillance may reveal neurological improvement in the individuals.

## Conclusions

This study demonstrated that traumatic dural tears in spine fractures can be suspected in patients who have preoperative neurological deficits, an increased interpedicular distance, severe canal encroachment, and a wide separation of laminar fractures. Surgeons should be able to identify a dural tear based on the existence of significant associative factors that indicate the likelihood of a traumatic dural tear. The data obtained from this study will enable them to enhance their operational planning and make early preparations for the management of dural tears.

## Appendices

Pro forma for data collection

Name/RN: _____________________

Hospital: _______________________

Section 1: Personal details and Demographics

1. Age: _________years old

2. Race: Malay [__] Chinese [__] Indian [__] Others [__]

3. Sex: Male [__] Female [__]

4. Mechanism of Injury: MVA [___] Fall from Height [___]Section 2: Clinical and Radiological Parameters

1. Diagnosis during admission: ___________________________

2. ASIA impairment scale (preoperative): A [__] B [__] C [__] D [__] E [__]

3. Fracture level:

• Upper Thoracic: ________

• Lower Thoracic: ________

• Thoracolumbar: ________

• Lumbar: ________

4. Fracture Morphological Pattern (based on AO Spine Injury Classification): ___________

5. Imaging findings:

• Interpedicular distance at the fracture level (by radiograph AP

view):______mm

i. Increment of the distance compare to cranial/caudal vertebral: ___mm

• Pedicle fracture (CT scan): YES [__] NO [__]

If, YES BILATERAL [__] UNILATERAL

[__]

• Ratio of central canal diameter at fracture level (CT scan): _______

*Neural canal encroachment: measured by the ratio of retropulsed bony

fragment anteroposterior diameter and central canal anteroposterior diameter in

CT axial plane**

• Laminar fracture (CT scan): YES [__] NO [__]

if YES, measure fracture gap:_____mm

• Facet joint fracture (CT scan): YES [__] NO [__]Section 3: Outcomes measures

a. Dural tear: YES [___] NO [___]

if YES, state length of dural tear: ______mm

Repair: [____] if repair, state method of repair:

________________

Not Repair: [____]

b. Volume of blood loss during surgery: ___________ml

c. Duration of surgery: __________ minutes/hour

d. ASIA impairment scale (postoperative in 3 months):

A [__] B [__] C [__] D [__] E [__]

Section 4: Complications

[____] Persitent CSF leak [____] Pseudomeningocele [____] Re surgery

[____] Meningitis [____] Wound breakdown [____] SSI

[____] Others: ______________
